# Resistant starch types 2 and 4 induce distinct and reversible changes in the human gut microbiome

**DOI:** 10.1128/spectrum.00763-26

**Published:** 2026-07-24

**Authors:** Elisa Piperni, Aitor Blanco-Míguez, Claudia Mengoni, Gianmarco Piccinno, Michal Punčochář, Jennifer Ren, Nicola Segata, Francesco Asnicar, Angela C. Poole

**Affiliations:** 1 Department CIBIO, University of Trento 19034, Trento, Italy; 2 Department of Experimental Oncology, IEO European Institute of Oncology IRCCS, Milan, Italy; 3 Division of Nutritional Sciences, Cornell University 5922, Ithaca, New York, USA; University of Nebraska-Lincoln, Lincoln, Nebraska, USA

**Keywords:** gut microbiome, resistant starch, metagenomics, amylases, CAZymes

## Abstract

Resistant starch (RS) can confer benefits for the gut microbiome and host cardiometabolic health. However, different types of resistant starch can differentially affect gut microbiome composition and functional capacity, especially given interindividual variability in responses, thus limiting the application of resistant starch in dietary strategies. We used shotgun metagenomics to perform a secondary analysis of samples collected during a previously reported randomized clinical trial to determine the effects of dietary supplementation with two types of resistant starch (RS2 and RS4) and a digestible starch (control) on the gut microbiome. Both resistant starch types induced distinct but transient alterations in the gut microbial community. RS2 enriched the keystone degrader, *Ruminococcus bromii*, and *Blautia glucerasea*, whereas RS4 favored *Parabacteroides distasonis* and known but uncharacterized microbial species such as a Lachnospiraceae bacterium. Moreover, we detected strain-level differences in the response of *Bifidobacterium adolescentis* to resistant starch. Microbial functional profiling revealed an enhanced capacity for complex carbohydrate utilization following resistant starch intake, including increased abundance of specific α-amylases, glycoside hydrolases, starch utilization systems, and other currently uncharacterized genes. Identifying the bacterial strains and genes that respond to different RS types will help to more accurately predict who will benefit from a given RS type. Our findings demonstrate that RS2 and RS4 differentially shape microbial ecology and metabolic capacity and provide a foundation for microbiome-informed personalization of resistant starch-based dietary interventions.

**IMPORTANCE:**

Dietary intake influences human health by modulating metabolism, partly by shaping the microbiota inhabiting the gut. Resistant starch (RS), a dietary fiber, is associated with metabolic improvements. While previous research has explored how RS alters the gut microbiome, RS comprises five types with differing physical and chemical characteristics, and the distinct impacts of each type on the microbiome and host health have not been fully characterized, particularly using high-resolution approaches such as shotgun metagenomics. In this secondary analysis of samples from a longitudinal crossover intervention study, we link dietary supplementation with RS2 and RS4 with distinct and transient changes in the composition and functional potential of the human gut microbiome. Specifically, we identify species that increase in abundance with each RS type, accompanied by increases in genes and pathways involved in complex carbohydrate utilization. The findings support the development of precision nutrition strategies utilizing RS supplementation to improve metabolic health.

This study is registered with ClinicalTrials.gov as NCT05743790.

## INTRODUCTION

Diet is a major determinant of human physiology and gut microbiome composition and functional capacity (1). Specific dietary components can interact directly with intestinal microbes, further influencing host health (2, 3). Among these, resistant starch (RS), comprising distinct forms of dietary fiber, is an important modulator of gut microbial ecology (4) and host metabolism (5, 6). RS escapes digestion by human enzymes in the small intestine and reaches the colon largely intact, where it undergoes bacterial fermentation (7). This process produces beneficial metabolites, notably short-chain fatty acids, which exert well-known positive effects on human health (8). Mounting evidence shows that RS consumption may reduce systemic inflammation and improve insulin sensitivity, glucose metabolism, and gut function (9–12). RS is classified into five types based on their physical and chemical properties: RS type 1 (starch physically encased in non-digestible structures, e.g., fibrous cell walls); RS type 2 (RS2, native granular starch with b-type crystallinity); RS type 3 (RS3, retrograded starch), RS type 4 (RS4, chemically modified starch), and RS type 5 (starch consisting of amylose-lipid complexes that form during food processing). While certain types (RS2, RS3, and RS4) are most frequently tested in human studies (13), the distinct impacts of different RS types on the gut microbiome and the resulting impact on host cardiometabolic outcomes remain unclear. In part, this gap results from challenges related to the wide variation in responses to RS across individuals, likely due to differences in baseline microbiome composition and host factors (such as habitual dietary fiber intake and genetics) (14). Advances in identifying microbiome-mediated mechanisms that underlie the metabolic benefits of RS are important to inform dietary strategies that leverage RS to promote metabolic health.

To date, most studies investigating how the gut microbiome responds to RS intake rely on 16S rRNA gene sequencing to show that different RS types induce distinct shifts in microbiome composition. These differential responses likely reflect how the unique physicochemical structure of each RS type determines microbial accessibility and substrate utilization, leading to the selective enrichment of specialized primary degraders and their cross-feeding partners (15). For instance, RS2 is commonly associated with an increase in starch-degrading Firmicutes, notably the keystone species *Ruminococcus bromii* (7, 16), and butyrate producers like *Eubacterium rectale* (16). In contrast, RS4 is reported to reduce Firmicutes relative abundance while favoring Bacteroidetes and Actinobacteria (17), including increases in species such as *Parabacteroides distasonis* and *Bifidobacterium adolescentis* (16). Despite these important taxonomic insights, the limited resolution of 16S rRNA gene surveys is often insufficient to detect changes at the species and strain levels (18, 19) , and unable to capture microbial gene functions, such as those encoding carbohydrate-active enzymes (CAZymes). These limitations preclude a precise understanding of microbiome remodeling upon RS supplementation. Understanding the impact of different RS types on gut microbiome composition and function is critical to addressing interindividual variability in response.

A single multiomics study integrated 16S rRNA gene sequencing with metaproteomics and metabolomics (4), revealing associations between high RS2 intake and pathways involved in lipid and glucose metabolism, as well as an increase in proteins related to energy production, nucleotide metabolism, and starch utilization. Two studies applied shotgun metagenomics to investigate RS-specific effects on the microbiome, but these focused exclusively on RS2 and involved small cohorts (12, 20). Therefore, a comprehensive, high-resolution characterization of how different RS types influence both microbiome composition and functional capacity is still needed to ultimately achieve the knowledge required to design precision nutrition protocols that recommend the dietary fiber sources providing the greatest beneficial impact on an individual basis.

In this secondary analysis of samples collected during a previously reported dietary intervention study (21), we use shotgun metagenomics to investigate the differential and shared effects of RS2 and RS4 supplementation, alongside a digestible starch control (Ctl), on the human gut microbiome. This approach allows for the detailed assessment of both taxonomic composition shifts and changes in functional potential (i.e., the microbial metabolic capacity), which could not be characterized in the previous analysis based on 16S rRNA gene sequence data. Moreover, the sample size included 68 healthy adults, which allowed us to evaluate conserved and interindividually variable microbiome responses to two different RS types easily obtained for research purposes.

## RESULTS

### Analysis of samples from the resistant starch supplementation study

**Fig 1:**
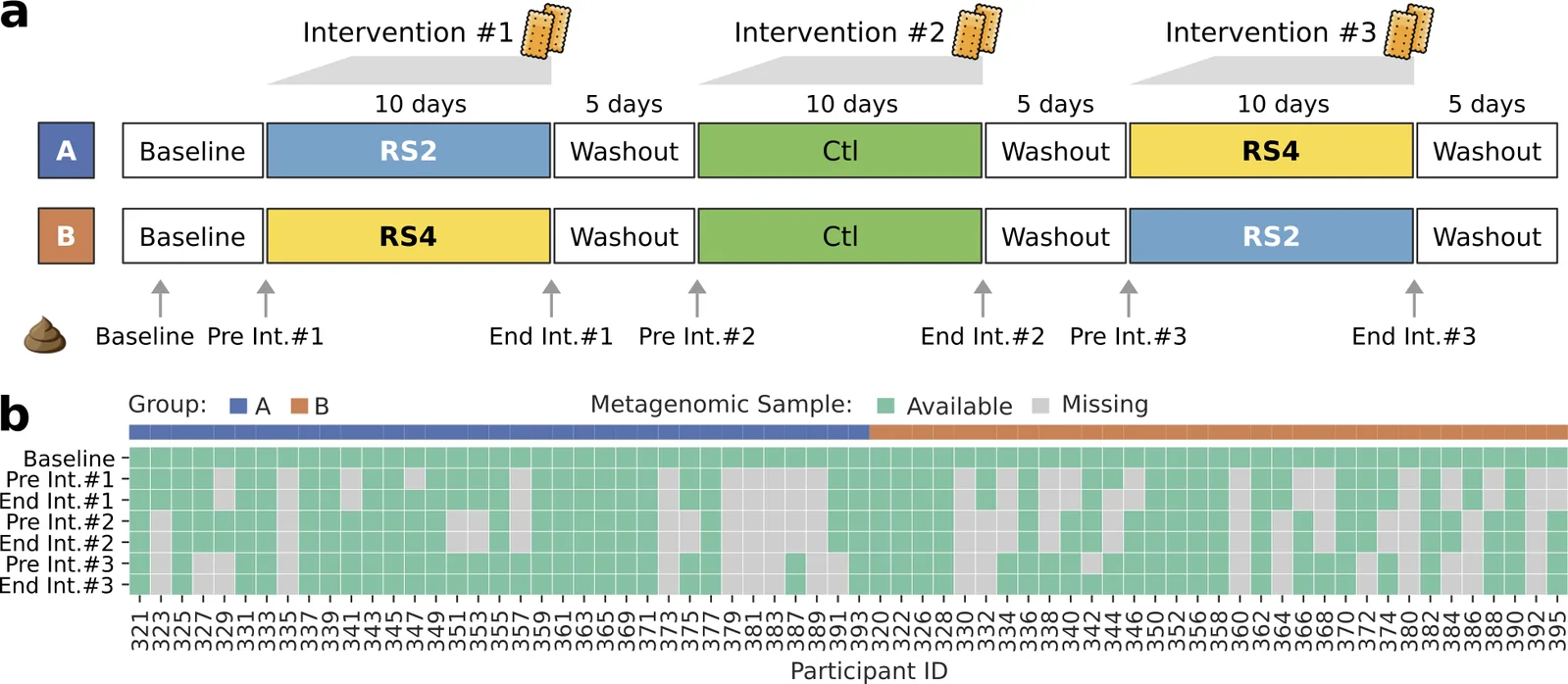
Overview of the study design and microbiome sampling. (**a**) Participants were randomly assigned to group A or B. Group A received the supplement crackers in the order RS2, Ctl, and RS4, while group B received them in the order RS4, Ctl, and RS2. Fecal samples were collected throughout the study: at baseline and before and after each intervention. (**b**) In total, 341 stool microbiome samples from 68 participants were analyzed. Participants from group A (*n* = 35, blue) and group B (*n* = 33, orange) provided a total of 180 and 161 samples, respectively. Ten participants provided samples only at baseline (six in A and four in B).

We performed a secondary analysis of samples collected during a dietary intervention study with a longitudinal, crossover design (21). Briefly, our analysis included stool samples collected from 68 participants, with 58 participants providing at least one sample in addition to baseline. During the study, participants supplemented their regular diet with crackers containing either RS2, RS4, or digestible starch (Ctl) for 10 days, with each intervention followed by a 5-day washout period. Subjects were randomized into two groups (A and B), which differed in supplementation order: group A received RS2, Ctl, and RS4, while group B received RS4, Ctl, and RS2. During the RS2 and RS4 interventions, the target amount of RS was 30 g per day, provided through a 120 g daily serving of crackers (Fig. 1a). There were no significant differences in body fat percentage and age between the two treatment groups (median body fat A = 23.7% and B = 23.9%, median age A = 26 years and B = 24 years) at baseline (Fig. S1a). Participants were asked to maintain their habitual physical activity and dietary intake (Fig. S1b). Fecal samples were collected at baseline and throughout the study, both before (Pre) and after (End) each intervention (Fig. 1a). We analyzed DNA from the fecal samples using shotgun metagenomic sequencing (Fig. 1b), followed by taxonomic and functional profiling.

### RS type 2 and 4 supplementation effects on gut microbiome composition

Microbiome composition significantly differed between groups at End Int. #1 and #3 (i.e., end of supplementation with RS; permutational analysis of variance (PERMANOVA) on Bray–Curtis dissimilarity *P* values = 2.97e-2 and 9.90e-3, respectively). No significant differences between the groups were observed at baseline, at any pre-intervention time points (Pre Int. #1–3, Fig. 1a), or at the post-control intervention time point (End Int. #2, Fig. S2a, Table S1). Furthermore, following RS interventions, we did not observe significant differences in α-diversity indices, with only a decrease in Shannon’s entropy (but not richness) after RS2 supplementation in group B (*P* value = 1.10e-2, Fig. S2b). These results indicate that RS2 and RS4 exert a divergent impact on gut microbiome composition but do not significantly affect its α-diversity.

When combining samples from both groups by intervention type, we observed significant microbiome composition differences between pre-intervention and end intervention for both RS types (PERMANOVA on Bray–Curtis dissimilarity *P* values = 4.95e-2 and 3.96e-2 for RS2 and RS4, respectively). The Ctl supplementation with digestible starch (Int. #2) did not elicit a significant change (PERMANOVA *P* value = 1, Fig. S2c, Table S1). Moreover, no significant differences were found between samples from before the interventions and those collected after the 5-day washout periods (Pre Int. #1 vs #2 and Pre Int. #1 vs #3, Table S1). Overall, these patterns indicate that washout periods were of sufficient length to revert the microbiome composition to the pre-intervention state, and that the end-intervention microbiome divergence between groups was due to temporary shifts in microbiome compositions induced by RS2 and RS4 supplementation.

### RS type-specific changes in relative abundance

**Fig 2:**
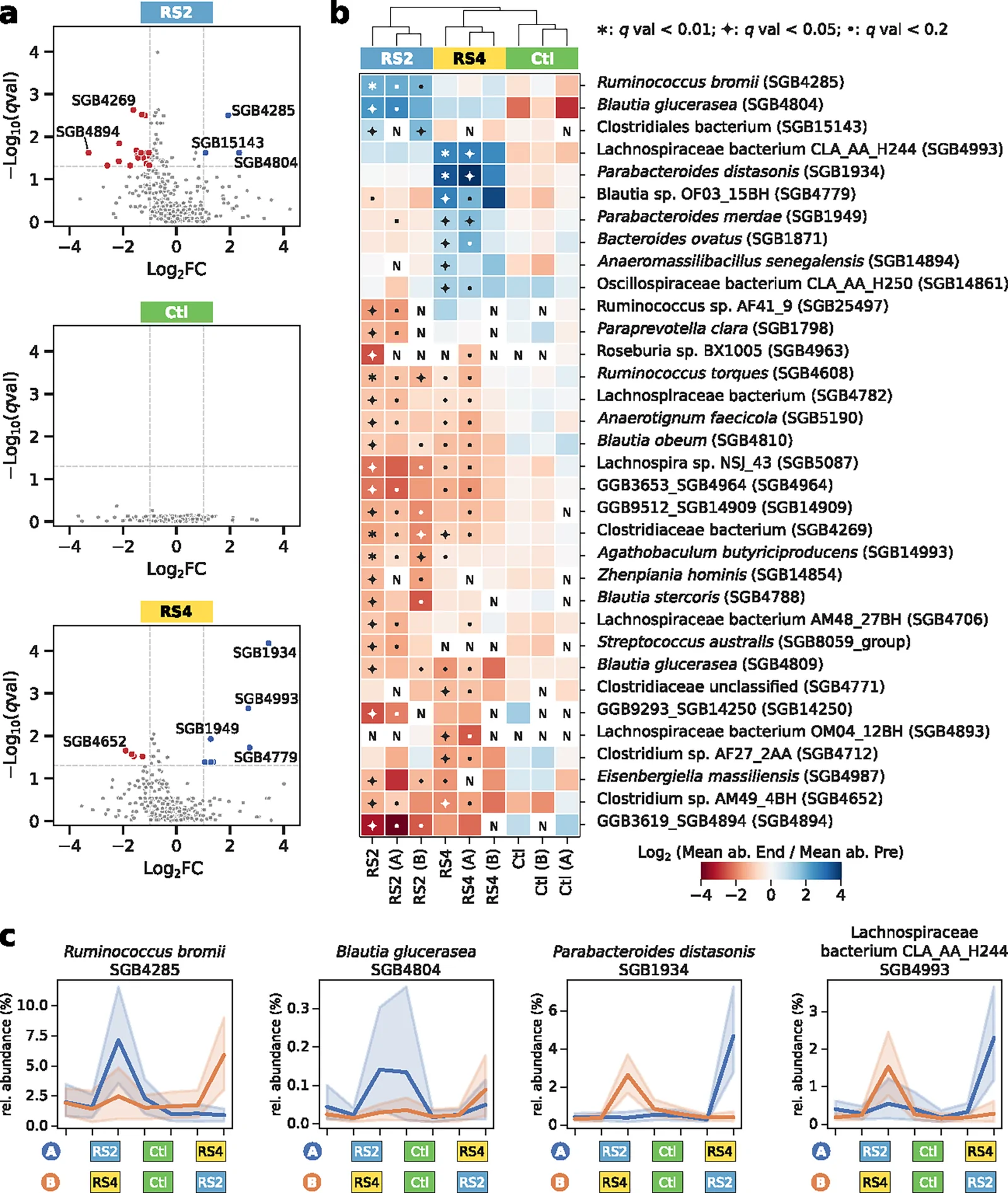
RS type 2 and 4 supplementation had distinct impacts on specific SGBs. (**a**) SGBs significantly increasing (blue) and decreasing (red) upon RS supplementation. The *x*-axis represents the log_2_ fold change (LFC) between the SGB mean relative abundance before and after each intervention, with positive values representing an increase in abundance and negative values representing a decrease. The vertical gray dotted lines mark the cutoff for SGBs with an abs(LFC) of >1. The *y*-axis is the log_10_ of the *q* values (Benjamini–Hochberg false discovery rate correction of *P* values from the Wilcoxon test). Dots above the horizontal gray dotted lines are significant SGBs with a *q* value of <0.05. (**b**) Changes in mean relative abundance for the 34 SGBs with abs(LFC) of >1 and *q* value of <0.05 in at least one intervention. While the increasing SGBs tend to show unique alterations across the two RS types, we observed for some SGBs a decrease in abundance regardless of the consumed RS type. Significance levels are marked as follows: ∗, *q* value <0.01; ✦, *q* value <0.05; •, *q* value <0.2. The blank cells with “N” represent SGBs that were not tested due to low abundance or prevalence (methods). (**c**) Mean relative abundance longitudinal patterns of the four SGBs with the strongest increase in relative abundance upon the two RS interventions (LFC >1.5 and lowest *q* values). The relative abundance values increase with RS supplementation while returning to baseline levels after the washout period. Individuals from group A are depicted in blue, while those from group B are in orange. Shaded areas represent the 95% confidence intervals. In the figure, all pre- and end-metagenomic samples are included with corresponding baseline samples.

We next analyzed the relative abundances of individual species-level genome bins (SGBs) before and after each intervention and defined differences based on log_2_ fold change (LFC) of mean relative abundance at End compared to Pre (Materials and Methods). While the intervention with digestible starch (Ctl) was not associated with changes in the relative abundance of any species (all *q* values >0.2), RS2 and RS4 intake led to a significant alteration in the abundance of 60 and 24 species, respectively (*q* < 0.05, Table S2). Among the most significantly increasing bacteria upon RS2 supplementation (LFC >1), we found *Ruminococcus bromii* (SGB4285: LFC = 1.9, *q* = 3.1e-3), *Blautia glucerasea* (SGB4804: LFC = 2.3, *q* = 2.4e-2), and a Clostridiales bacterium (SGB15143: LFC = 1.1, *q* = 2.4e-2; Fig. 2a). On the other hand, RS4 supplementation elicited a significant increase in the relative abundance of *Parabacteroides distasonis* (SGB1934: LFC = 3.4, *q* = 6.6e-5), Lachnospiraceae bacterium CLA AA H244 (SGB4993: LFC = 2.7, *q* = 2.3e-3), *Parabacteroides merdae* (SGB1949: LFC = 1.3, *q* = 1.1e-2), *Blautia* sp. OF03 15BH (SGB4779: LFC = 2.7, *q* = 1.9e-2), *Bacteroides ovatus* (SGB1871: LFC = 1.1, *q* = 4.1e-2), Oscillospiraceae bacterium CLA AA H250 (SGB14861: LFC = 1.4, *q* = 4.1e-2), and *Anaeromassilibacillus senegalensis* (SGB14894: LFC = 1.3, *q* = 4.1e-2; Fig. 2a). These results were consistent when the two intervention groups were considered separately (Fig. 2b; Table S3, Fig. S3a).

The abundance of the increased SGBs returned to baseline levels at the end of the 5-day washouts (Pre Int. #2 and Pre Int. #3; metagenomes were not available at the end of the washout after End Int. #3; Fig. 2c), indicating that the taxonomic response to RS is generally reversible (20). To account for the compositionality of these data, we also analyzed SGB prevalences and found that they were largely unaffected by RS supplementation (e.g., *R. bromii* prevalence = 44.7% at Pre RS2 and = 48.9% at End RS2, *P. distasonis* prevalence = 90.9% both at Pre RS4 and End RS4; Table S2), indicating that either (i) few participants lost or gained these species throughout the study period or (ii) these species were below the limit of detection due to the sampling or sequencing approach.

We also identified 21 SGBs with a significant decrease (LFC <−1 and *q* value <0.05) in relative abundance after RS2 supplementation (Table S2). Of these, 17 represented SGBs from known species, including *Ruminococcus torques* (SGB4608: LFC = −1.29, *q* = 3.0e-3), *Lachnospira* sp. NSJ_43 (SGB5087: LFC = −2.15, *q* = 1.4e-2), and three *Blautia* species: *Blautia obeum* (SGB4810: LFC = −1.03, *q* = 2.4e-2), *Blautia stercoris* (SGB4788: LFC = −1.45, *q* = 2.8e-2), and *B. glucerasea* (SGB4809: LFC = −1.12, *q* = 4.3e-2). The remaining four unknown SGBs (uSGBs; i.e., species not yet isolated and phenotypically characterized) were all assigned to the Firmicutes phylum, with two further classified into the Lachnospiraceae family (SGB4964 and SGB4894, Fig. 2b). Interestingly, two distinct SGBs assigned to the *B. glucerasea* species exhibited significant and opposite responses to RS2 supplementation; SGB4804 was increased, whereas SGB4809 was decreased (Fig. 2b and c). This divergence could potentially suggest SGB-specific metabolic differences influencing RS2 utilization capabilities. On the other hand, RS4 supplementation led to a significant decrease of four SGBs, including two *Clostridium* species (SGB4652: LFC = −1.90, *q* = 2.2e-2, and SGB4712: LFC = −1.66, *q* = 2.7e-2) and one Lachnospiraceae bacterium (SGB4893: LFC = −1.60, *q* = 3.0e-2), as well as one uSGB (ID: 4771: LFC = −1.27, *q* = 3.0e-2; Fig. 2b; Table S2). Overall, RS2 and RS4 showed distinct impacts on specific SGBs. However, effects on SGBs that decreased in abundance were less RS-type specific than those on SGBs that increased, with several SGBs exhibiting reduced abundance regardless of the RS type consumed (Fig. 2b; Fig. S3b, and Table S2).

### Strain-level interindividual variability in response to RS intake

There is considerable interindividual variability in the effects of dietary fiber supplementation, including RS, on the human gut microbiome (14, 16). Furthermore, only some strains of certain bacterial species, such as *Bifidobacterium adolescentis*, are capable of utilizing and growing on starches *in vitro* (22–24). Thus, we investigated whether individual responses to RS supplementation could be attributed to strain-level differences within SGBs. We first confirmed that samples from study participants generally maintained the same strains across longitudinal time points (Fig. S4a and b). As expected, SGBs that significantly increased in overall abundance after RS2 or RS4 supplementation (Fig. 2b) exhibited high intersubject consistency in their response (Fig. S4c).

We then identified 36 SGBs that showed a mixed response to RS intake (i.e., no significant change in overall relative abundance but high variability in individual fold changes; RS2 *n* = 24, RS4 *n* = 21, with *n* = 9 in common). However, we could not detect significant links between the strain-level phylogenetic structure of such SGBs and responses to RS2 or RS4 (Table S4), which may be due to the relatively modest sample size and a possible decoupling between functional properties of strains and the phylogenetic relatedness that we inferred. To expand the phylogenetic characterization for *B. adolescentis* (SGB17244), we included seven genomes whose RS-degrading activities were tested *in vitro* (23). Although no clear relationship emerged between phylogenetic distance and RS responsiveness, strains from subjects with increased *B. adolescentis* abundance after RS supplementation broadly tended to cluster together (Fig. S4d).

We also investigated whether variability in RS response was associated with the genomic carriage of certain CAZymes (Materials and Methods). Profiling of *B. adolescentis* genomes suggested a potential association with the presence of certain carbohydrate-binding module families (CBM25, CBM26, and CBM74), which were detected exclusively in strains reported to degrade RS *in vitro* (23) and have been reported to have starch-binding activity (25) (Table S5). However, no consistent patterns were observed in the reconstructed metagenomic-assembled genomes (MAGs), as these CBMs were present also in MAGs derived from subjects whose *B. adolescentis* decreased following RS intervention. This lack of clear association may reflect the predominantly medium quality (MQ) of the reconstructed MAGs (completeness 50%–90%, contamination <5%), which could limit the sensitivity of downstream analyses.

### Functional remodeling of the gut microbiome by RS supplementation

**Fig 3:**
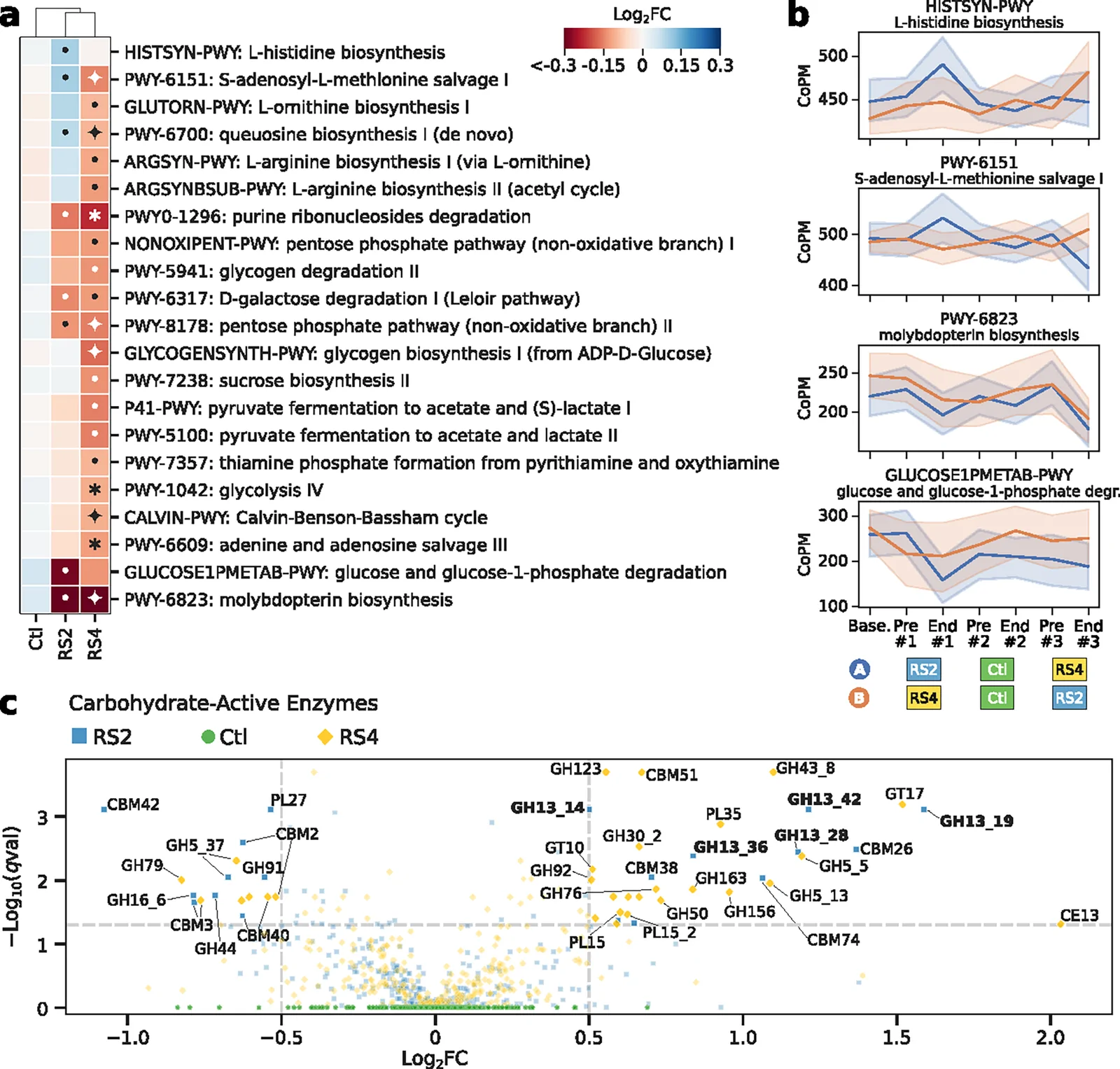
RS2 and RS4 supplementation affect the microbiome functional potential. (**a**) Changes in mean relative abundance of the 21 pathways with abs(log_2_ [Mean ab. End / Mean ab. Pre]) >0.1 and *q* value <0.2 in at least one intervention. Values from group A and group B are averaged together (Table S6). Significance levels are marked as follows: ∗, *q* value <0.01; ✦, *q* value <0.05; •, *q* value <0.2. While some pathways decreased after each intervention (i.e., PWY-6823, PWY-6317, PWY-8178, and PWY0-1296), others (such as PWY-6151, GLUTORN-PWY, and PWY-6700) were more abundant upon RS2 but reduced with RS4. (**b**) Mean relative abundance (measured in copies per million [CoPM]) across the study time points of the four pathways that were more strongly affected by the two RS interventions. Shaded areas represent the 95% CI. Different longitudinal patterns emerged: HISTSYN-PWY was significantly increased by RS2 (spikes at End Int. #1 in group A and at End Int. #3 in group B) but largely unaffected by RS4; PWY-6151 showed an increase upon RS2 but a drop upon RS4; PWY-6823 was significantly decreased by both interventions; and GLUCOSE1PMETAB-PWY was reduced only in group A for both RS2 and RS4. Group A is depicted in blue (RS2 at Int. #1 and RS4 at Int. #3), while group B is in orange (RS2 at Int. #3 and RS4 at Int. #1). In the figure, all pre- and end-metagenomic samples are included with corresponding baseline samples. (**c**) Fold change and significance of the CAZymes across interventions (RS2, blue squares; Ctl, green dots; RS4, yellow diamond). While no significant differences were observed for the control intervention, both RS2 and RS4 supplementation were associated with significant alterations in the abundance of specific CAZyme subfamilies (Table S9). Subfamilies of glycoside hydrolase 13 are highlighted in bold. The dashed horizontal line marks *q* value <0.05, while vertical lines mark abs(log_2_FC) >0.5.

Differential abundance analysis of gut microbiome functional potential profiles revealed both shared and unique alterations in microbial metabolic pathways upon the two RS interventions (Fig. 3a; Table S6). Four pathways were significantly decreased by either RS type (*q* value <0.2): molybdopterin biosynthesis (PWY-6823: RS2 *q* = 8.1e-2, RS4 *q* = 1.1e-2), D-galactose degradation (PWY-6317: RS2 *q* = 8.1e-2, RS4 *q* = 7.5e-2), pentose phosphate pathway (PPP; PWY-8178: RS2 *q* = 1.4e-1, RS4 *q* = 3.2e-2), and purine ribonucleoside degradation (PWY0-1296: RS2 *q* = 1.4e-1, RS4 *q* = 8.8e-3). Additionally, in group A for both RS types, the glucose and glucose-1-phosphate degradation pathway (GLUCOSE1PMETAB-PWY) was reduced (Table S7). These changes suggest a potential shift away from simple sugar utilization (i.e., galactose and glucose), possibly in favor of fermentation of complex starches, as well as a reduction in cofactor biosynthesis (i.e., molybdopterin) and nucleotide turnover (i.e., PPP and purine degradation). Upon RS4 supplementation, we also observed a reduction in glycogen and sucrose biosynthesis pathways (GLYCOGENSYNTH-PWY and PWY-7238: *q* = 3.4e-2 and *q* = 6.9e-2) and in the glycogen degradation pathway (PWY-5941: *q* = 7.5e-2), likely indicating a decrease in the proportion of microbial species relying on internal polysaccharide reserves as an energy source. We also observed a decrease of glycolysis IV (PWY-1042: *q* = 8.8e-3) and, in group A, of pyruvate fermentation to acetate and lactate pathways (P41-PWY and PWY-5100: *q* = 9.3e-2). No pathways were significantly increased upon RS4 intake. In contrast, RS2 supplementation was significantly associated with enrichment of L-histidine biosynthesis (HISTSYN-PWY: *q* = 1.1e-1), potentially reflecting the expansion of taxa capable of degrading RS2. Additionally, the S-adenosyl-L-methionine salvage (PWY-6151: *q* = 1.1e-1) and queuosine biosynthesis (PWY-6700: *q* = 1.1e-1) pathways were more abundant with RS2 but reduced with RS4 (Fig. 3b). Finally, supplementation with digestible starch (Ctl) did not result in any significant pathway alterations (Table S6).

In terms of gene families, RS2 and RS4 interventions led to 14,935 and 10,557 significantly altered UniRef50 clusters (26), respectively (*q* value < 0.05, Materials and Methods; Fig. S5a and b). To simplify interpretation, we focused on gene families with high absolute fold changes and a lower *q*-value threshold, resulting in 1,134 increased and 112 decreased genes upon RS2 supplementation and 1,741 increased and 43 decreased genes upon RS4 [*q* value <0.01 and abs(log_2_FC) >1, Table S8]. Consistent with the pathway-level findings, an α-galactosidase (UR50_A0A176U5G5) was less abundant after RS supplementation, albeit only significant for RS2. Among increased genes, RS4 intake was associated with an enrichment of 31 starch utilization system (Sus) genes (out of *n* = 530 tested), including 12 SusC and 19 SusD protein families. These genes are associated with polysaccharide utilization loci, some of which are involved in starch degradation. We also detected an increase in two genes encoding starch-binding proteins (UniRef50_A0A074L0B8 and UniRef50_A0A1G6G9R4). Furthermore, after RS4 supplementation, we detected significantly increased abundance in one glycosidase (*n* = 33), seven glycoside hydrolases (GHs) (*n* = 142), one α-amylase (*n* = 57), and an isoamylase gene (*n* = 1). On the other hand, RS2 supplementation led to a significant increase of two α-amylases (*n* = 59), one maltogenic amylase (*n* = 1), five glycosidases (*n* = 36), and four pullulanases (*n* = 24) (27). Notably, a large fraction of the significantly increased UniRef50 lacked a functional description (34.7% or 393 of 1,134 for RS2, and 50.4% or 878 of 1,741 for RS4; Materials and Methods) indicating the presence of many uncharacterized gene families that could have a role in RS metabolism (Table S8).

To further investigate the microbiome’s functional potential for RS utilization, we focused on CAZymes (Materials and Methods) (25). While the control intervention did not induce significant changes, both RS2 and RS4 supplementation were associated with significant alterations in the abundance of specific CAZyme subfamilies. RS2 supplementation resulted in 10 significantly more abundant and 9 less abundant CAZyme subfamilies, while RS4 resulted in 22 more abundant and 7 less abundant ones (Fig. 3c; Table S9).

Specifically, RS2 intake was associated with increased abundance of three carbohydrate-binding modules (CBM26, CBM38, and CBM74) and seven GHs, including GH55, GH158, and five distinct CAZy subfamilies of GH13, indicating an enhanced capacity of the microbiome for starch binding and degradation. In contrast, RS4 supplementation led to a broader and more diverse increase in CAZymes compared with RS2, including 13 GHs and CBM51, as well as 5 polysaccharide lyases (PL12, PL12_2, and PL15_2, putatively targeting heparin and heparan sulfate, and PL35 and PL29, with chondroitin as a substrate), 2 glycosyltransferases (GT10 and GT17), and 1 carbohydrate esterase (CE13, list of putative substrates in Table S9). Notably, six of the enriched CAZy families were also identified using the approach employed for the pathway and gene families analyses, despite its lower specificity for CAZyme profiling (Materials and Methods, Fig. S5c). Among the decreased CAZymes, both RS2 and RS4 supplementation were associated with reduced abundance of CBM2, CBM3, CBM40, and GH5_37. In addition, RS4 supplementation specifically reduced GH79 and GH166, whereas RS2 supplementation reduced GH16_6, GH44, and GH91.

## DISCUSSION

Our analysis indicates strong evidence that dietary supplementation with RS2 and RS4 distinctly shapes the human gut microbiome composition and functional potential, reflecting their unique physicochemical and morphological properties (13). Notably, RS2 (native starch) increased the relative abundance of *Ruminococcus bromii*, a known keystone species for RS degradation (7) due to its specialized amylolytic machinery (28). In contrast, RS4 (chemically modified starch) promoted species such as *Parabacteroides distasonis* and *Bacteroides ovatus*. While these species were previously reported to increase in relative abundance in response to RS (16, 17), our sample size allowed us to identify novel species linked to RS intake. For instance, *Blautia glucerasea* (SGB4804) increased in relative abundance after RS2 supplementation, while other *Blautia* species decreased.

The use of shotgun metagenomics allowed us to resolve taxonomy to the strain level and to profile functional microbiome potential, which was not possible from the 16S rRNA gene amplicon data from our previous study (21). We identified 37 taxa at the species level that changed during the RS2 treatment in this analysis, compared to 9 taxa at the species level in our previous analysis. We identified 13 taxa at the species level that changed during the RS4 treatment in this study, compared to 4 taxa at the species level in our previous analysis. Both analyses identified significant changes in the relevant abundances of *Ruminococcus bromii*, *Faecalibacterium prausnitzii*, *Blautia obeum*, *Dorea formicigenerans*, and *Ruminococcus torques* in response to RS2, and both analyses identified significant changes in the relevant abundances of *Parabacteroides distasonis*, *Bacteroides ovatus*, and *Dorea formicigenerans* in response to RS4. In both analyses, no taxon significantly changed in relative abundance during the control treatment. We also identified currently uncharacterized species, such as Clostridiales bacterium (SGB15143) and Lachnospiraceae bacterium (SGB4993), which increased with RS2 and RS4 supplementation, respectively. These findings suggest that additional, currently uncharacterized species may be involved in RS utilization. Additionally, due to the longitudinal and crossover study design, we observed that the effects of RS supplementation were transient, with microbiome composition returning to a pre-intervention state following 5-day washout periods. We also observed strain persistence throughout the intervention period and detected strain-level interindividual variability of *B. adolescentis* in response to RS supplementation (23, 29).

Supplementation with RS2 and RS4 also altered the gut microbiome’s functional potential. We observed changes in gene and pathway abundances, characterized by a reduction in simple sugar utilization and an increase in complex carbohydrate degradation. We also found significant abundance variations in specific CAZyme families upon both the RS2 and RS4 interventions. In particular, GH13 was found among the top families being affected by RS2, with five subfamilies significantly more abundant at the end of the intervention. GH13 and CBM26, which also increased in response to RS2, are known to be utilized by *Ruminococcus bromii* to break down starch (30). Conversely, while RS4 intake did not significantly affect GH13 levels, it was associated with an increase in different CAZyme families, including various glycoside hydrolases and polysaccharide lyases. Some CAZyme families decreased in abundance in response to both RS2 and RS4 (GH5_37, CBM2, CBM3, and CBM40) and may represent taxa that are displaced during RS fermentation or functions that are less important when RS substrate is present. Overall, the response to RS2 appears to be more specialized than the response to RS4, which increased the abundance of a greater number and diversity of CAZyme families. These findings indicate changes in the gut microbiome’s ability to access and metabolize RS and further support the distinct effects of the two RS types (31). Nonetheless, because our results are based on metagenomic data, they capture functional potential rather than activity and may largely represent changes in microbiome composition, including the increased abundance of taxa carrying these functions (e.g., *Bacteroides* spp.). Metatranscriptomic analyses would be required to determine whether these patterns are accompanied by gene expression changes indicative of true metabolic flexibility of the community. Additionally, we identified several uncharacterized genes whose abundances were significantly altered, suggesting that this “microbial functional dark matter” (32) may be involved in currently undescribed mechanisms of RS degradation or downstream metabolic effects and deserves further exploration.

Our analysis has some limitations. First, an even larger sample size may have increased our statistical power to detect additional associations and strain-level patterns. Second, the study cohort is geographically homogeneous, which may restrict the generalizability of our findings, as it does not fully capture microbiome variability. Populations from different areas of the globe harbor different strains of fiber-degrading bacteria, such as *Prevotella copri* (33, 34). Moreover, the limited duration of the study and the absence of data from samples collected after the second RS intervention prevent a complete assessment of the long-term reversibility and stability of the observed microbiome changes. Additional studies involving larger cohorts encompassing more diverse populations are therefore needed to validate and extend our findings. Moreover, future research should employ complementary approaches, such as *in vitro* assays, to establish underlying mechanistic links and incorporate multiomics data measuring host metabolic health responses to provide deeper insights (e.g., metatranscriptomics and metabolomics). Exploring the role of host genetic factors, such as the salivary amylase gene (*AMY1*), and accounting for differences in dietary habits, would also be crucial to fully understand the interindividual variability in responses to RS types. Finally, our results underscore the importance of moving beyond species-level resolution, indicating that targeting a higher sequencing depth would enable broader strain-level characterization.

In conclusion, our analysis shows that different types of RS elicit distinct changes in the composition and functional potential of the gut microbiome. We highlight the critical roles of microbial species diversity in shaping the response. Furthermore, the identification of unknown species and uncharacterized genes with metabolic functions related to starch degradation warrants further investigation. These findings collectively provide evidence to support the development of personalized nutrition strategies involving RS types, with the goal of modulating the gut microbiome to benefit host health. Characterizing microbiota response to different RS types with strain-level resolution could contribute to more accurate predictions of how individuals will respond. Coupling these data with measurements of changes in microbial metabolites, including bile acids, sterols, and short-chain fatty acids, and changes in metabolic health parameters will guide the development of personalized dietary fiber recommendations to prevent and manage conditions such as type 2 diabetes and hypercholesterolemia.

## MATERIALS AND METHODS

### Cohort description and study design

Biological samples used in this analysis were collected from 68 healthy adults enrolled in a 7-week open-label crossover dietary intervention conducted between October and December 2020 (clinicaltrials.gov ID: NCT05743790). We previously reported on the trial design and results under CONSORT guidelines (21). Briefly, participants were healthy males and healthy, non-pregnant or lactating females aged 18–59 years old. Exclusion criteria included a history of gastrointestinal diseases or surgery; diabetes, pre-diabetes, or impaired glucose tolerance; self-reported untreated thyroid condition; use of antibiotics 6 months before the start of the study; and chronic alcohol intake (>5 drinks/day).

Participants were assigned study identification (ID) numbers based on the order of enrollment; those with an odd ID number were allocated to group A, while those with an even ID number were allocated to group B. A single participant with an odd ID number was allocated to group B to balance the groups (ID: 395). Participants consumed crackers containing RS2 (HI-MAIZE 260 starch, Ingredion), RS4 (VERSAFIBE 1490 starch, Ingredion), or a digestible Ctl starch (AMIOCA TF starch, Ingredion) each for 10 days, in addition to their habitual diet. The groups received treatments in the following order: group A received RS2, Ctl, and then RS4; and group B received RS4, Ctl, and then RS2. During RS2 and RS4 treatments, the target dose of each type of starch was 30 g in a daily serving of 120 g of crackers. The actual amount in the baked RS2 crackers was 21.27 g per serving, and presumably a similar reduction occurred in the RS4 crackers, as previously reported (21). To minimize gastrointestinal discomfort, there was a 3-day ramp-up period, followed by 7 days at the full dose. Specifically, participants consumed 25% of the final dose on day 1, 50% on day 2, 75% on day 3, and 100% (120 g) from days 4 to 10. The RS2, RS4, and control crackers were approximately matched for total carbohydrate, fat, and protein content, but the control cracker contained more calories. Each treatment was separated by 5-day washout periods during which no study crackers were consumed. Participants collected fecal samples before (“Pre RS2,” “Pre Ctl,” or “Pre RS4”) and at the end (“End RS2,” “End Ctl,” or “End RS4”) of the treatment periods. Samples were stored at −80°C until lyophilized. After lyophilization, DNA was extracted from the samples for a 16S rRNA gene survey. The DNA was stored at −80°C until we prepared metagenomic libraries.

### DNA extraction and sequencing

We used the Illumina DNA Prep kit and IDT for Illumina DNA/RNA UD Index sets A, B, C, and D to prepare shotgun metagenomic libraries from lyophilized stool samples following the manufacturer’s protocol, using 100–500 ng of genomic DNA input for each sample, aiming for 300–650 bp average fragment sizes. We performed an additional right-side SPB cleanup using either a 0.53× or 0.6× ratio for libraries with high enough yields that exceeded average fragment sizes of 700 bp. We normalized all libraries to 8 nM before pooling. After pooling, we performed an additional left-side 0.7× SPB cleanup to remove <300 bp fragments, and the average fragment size of the final pool was 573 bp. We performed 2 × 150 bp paired-end sequencing using all four lanes of a NovaSeq 6000 S4 flow cell.

### Metagenomic sample pre-processing and quality control

Raw sequenced data were pre-processed and quality controlled using the pipeline available at https://github.com/SegataLab/preprocessing, which entails three steps: (i) removal of low-quality reads (*Q* < 20) and reads shorter than 75 nt or containing more than two ambiguous bases; (ii) removal of host contaminant DNAs (Illumina’s spike-in PhiX 174 and human genome, hg19); and (iii) synchronization of paired-end and unpaired reads. After pre-processing, the median number of reads was 66.29 million.

### Taxonomic and functional profiling

The shotgun metagenomic samples have been profiled within the bioBakery computational environment (35). To account for differences in sequencing depth, metagenomes were subsampled at 65 million reads (which was the median number of reads before subsampling). We employed MetaPhlAn 4 (Metagenomic Phylogenetic Analysis [36], v4.1, with the database vJun23_CHOCOPhlAnSGB_202307 and default parameters) for species-level taxonomic profiling and quantification of relative microbial abundances. For the functional potential profiling and the analysis of microbial gene families and pathways, we employed HMP Unified Metabolic Analysis Network (HUMAnN, v3.9) (35). For the profile and substrate annotations of CAZyme families, we employed Cayman (v0.10.3, https://doi.org/10.1038/s41564-026-02318-2) (command line: “cayman profile GMGC10.human-gut.95nr.no-rare.0.5.percent.prevalence_all_v3_FINAL.csv GMGC10.human-gut −1 {SAMPLE_R1.fastq} −2 {SAMPLE_R2.fastq} --orphans {SAMPLE_UN.fastq}).

### Strain-level phylogenetic reconstruction

Strain-level characterization of SGBs was performed with StrainPhlAn 4 (v4.1.0) with default parameters (37). Resulting phylogenetic trees were re-rooted at midpoint with Archaeopteryx (http://www.phylosoft.org/archaeopteryx) (38) and annotated and visualized with GraPhlAn (v1.1.3, available at https://github.com/biobakery/graphlan) (39). The Python library Dendropy was used for phylogenetic computing (40).

For *Bifidobacterium adolescentis* (SGB17244), we also built a phylogenetic tree that included reference genomes from strains previously tested for RS utilization *in vitro*. From literature, we identified nine such strains: P2P3, L2-32, 22L, 703B, DSM20083, DSM20086, DSM20087, DSM24849, and NCFB2229 (15, 23, 24, 41), eight of which have an available genome sequence in NCBI (P2P3 = GCA_003856735, L2−32 = GCA_000154085, 22L = GCA_000737885, 703 = GCA_002108035, DSM20083 [ATCC 15703] = GCA_000010425, DSM20086 = GCA_025289145, DSM20087 = GCA_000702865, and DSM24849 = GCA_000741415). Strains P2P3, L2−32, 22L, and DSM24849 have been reported to utilize RS; strains 703B, DSM20086, and DSM20083 do not use it, while DSM20087 exhibited low RS utilization. Five of these genomes (GCA_003856735, GCA_000154085, GCA_000737885, GCA_002108035, and GCA_000741415) are included in the MetaRefSGB database (vJun23), while three were not (GCA_000702865, GCA_000010425, and GCA_025289145). Because MetaRefSGB applies de-replication to avoid redundancy, genomes highly similar to those already present are excluded. In particular, GCA_000702865 is very close to GCA_002108155 (MASH distance = 0.00020368), while both GCA_000010425 and GCA_025289145 are very close to GCA_002108135 (MASH distances 0.000136297 and 0.000119496, respectively). Based on this, we included in the phylogenetic tree the five previously mentioned genomes and the two additional non-redundant genomes.

### Metagenomic assembly

Metagenomic assembly was performed with MEGAHIT v1.2.9 and included removal of contigs shorter than 1,000 bp and alignment of the remaining contigs against original raw data with Bowtie2 (v2.3.5) to obtain coverage information. Binning of the contigs was performed with MetaBAT (v2.12.1), and resulting putative MAGs were quality controlled with CheckM2. For downstream analyses, we retained high-quality (completeness >90% and contamination <5%, respectively) and MQ (completeness ≥50% and contamination <5%) MAGs. Taxonomic assignment was performed with PhyloPhlAn 3.0 (https://doi.org/10.1038/s41467-020-16366-7), resulting in the recovery of 83 MAGs assigned to *B. adolescentis* (SGB17244).

### *B. adolescentis* genomes and MAG annotation

These 83 MAGs, together with 7 *B. adolescentis* reference genomes previously tested *in vitro* for RS utilization and with available non-redundant genome sequences (GCA_000737885, GCA_003856735, GCA_002108035, GCA_002108155, GCA_000154085, GCA_000741415, and GCA_002108135; see Strain-Level Phylogenetic Reconstruction) were annotated using dbCAN2 (https://doi.org/10.1093/nar/gky418). For each genome, we retained only hits consistently identified by all three dbCAN annotation tools (HMMER, eCAMI, and DIAMOND). Then, for each CAZyme family, we counted the number of contigs with reported hits by at least two tools. To investigate whether the carriage or number of specific CAZymes was associated with response to RS interventions, we focused on the genomes underlying Fig. S4d, including reference strains and MAGs reconstructed from either “Pre” or “End” samples, thereby enabling assessment of responses to the interventions.

### Statistical analyses

#### α- and β-diversity and PERMANOVA

α- and β-diversities were computed with the script calculate_diversity.R from MetaPhlAn, with the MetaPhlAn profiles subsampled at 65 million reads used as input. A PERMANOVA was performed with the Bray–Curtis similarity index using the function adonis2 of the package vegan, with 100 permutations.

#### Identification of SGBs affected by RS supplementation

First, we analyzed samples from group A and B together, and for each intervention, we considered subjects with both Pre and End samples available (RS2, *n* = 47; Ctl, *n* = 44; and RS4, *n* = 44) and tested the species detected in at least five subjects and with at least 0.01% mean abundance either at Pre or End or at both time points (RS2, *n* = 359; Ctl, *n* = 373; and RS4, *n* = 361). Specifically, we performed the arcsin square-root transformation of the relative abundances of each SGB, and we applied Wilcoxon signed-rank tests between paired samples using the function wilcoxon from the scipy Python library (42). We then calculated the fold change as the log_2_ of the ratio between the mean relative abundance at End and the mean relative abundance at Pre (LFC). Nominal *P* values were corrected for multiple hypothesis testing using the Benjamini–Hochberg method with the function fdrcorrection from the statsmodels Python library (43). False discovery rate (FDR)-corrected *P* values were reported as *q* values. We focused on SGBs with *q* values <0.05 and abs(LFC) >1 (Fig. 2a; Table S2). Then, we tested the two groups separately (Table S3 , with the same criteria to select subjects and SGBs (group A: subjects RS2 *n* = 24, Ctl *n* = 23, RS4 *n* = 25; tested SGBs: RS2 *n* = 278, Ctl *n* = 282, RS4 *n* = 289; group B: subjects RS2 *n* = 23, Ctl *n* = 21, RS4 *n* = 19; tested SGBs: RS2 *n* = 243, Ctl *n* = 245, RS4 *n* = 222).

#### Identification of strain-level patterns in response to RS supplementation

We analyzed samples from group A and B together, and for each intervention, we considered subjects with both Pre and End samples available (RS2, *n* = 47, and RS4, *n* = 44) and tested the species detected in at least 20 subjects and with 0.05% mean abundance either at Pre or End or at both time points (RS2, *n* = 111, and RS4, *n* = 107, higher thresholds from the SGB-level analysis to ensure robustness at the strain level). We then focused on the SGBs not affected by the RS interventions, with *q* value >0.1 and −0.5 < LFC < 0.5 (RS2, *n* = 49, and RS4, *n* = 58). Of them, we calculated the LFC in each subject and considered the SGBs whose LFCs had an interquartile range of >2 and with the lower quartile <0 and upper quartile >0, denoting SGBs with high variability in response to RS across different subjects (RS2, *n* = 24, and RS4, *n* = 21, of which *n* = 9 in common). For these 36 SGBs, we built phylogenetic trees with StrainPhlAn using default parameters (37).

To evaluate the association between strain-level stratification and response to RS intake, we applied phylogenetically generalized linear models on the StrainPhlAn trees using anpan (Analyses of Microbial Phylogenies and Genes, v0.3.0; https://github.com/biobakery/anpan). Phylogenetic generalized linear mixed models are probabilistic models that account for the phylogenetic structure by encoding the tree structure as a correlation matrix. Specifically, we fitted a model in which the binarized LFC (i.e., increasing or decreasing in abundance upon RS intake) was the dependent variable. We considered only one of either Pre or End sample for each subject in each SGB-RS intervention pair of interest (with priority to the Pre sample when available in the StrainPhlAn tree). To select models with evidence of an existing phylogenetic effect, we selected models where expected log predictive density difference (ELPD) over a base GLM was greater than 4 and where the confidence interval (ELPD ± 2 × SE) did not include the value of 0. ELPD is a model comparison metric similar to the Akaike information criterion (Table S4).

#### Identification of pathways affected by RS supplementation

To account for differences in sequencing depth, we normalized the pathway abundances provided by HUMAnN with copies per million (CoPM), in which samples are constrained to sum to 1 million. We analyzed samples from group A and B together, and for each intervention, we considered subjects with both Pre and End samples available (RS2, *n* = 47; Ctl, *n* = 44; and RS4, *n* = 44). To test pathways, we considered both their abundance (in CoPM) and coverage (which is calculated by estimating the fraction of genes in a pathway based on their individual abundances and provides a confidence of the pathway being present in a sample, with values between 0 and 1). We tested the pathways with coverage greater than 0.8 in at least 60% of the samples, at either or both Pre and End time points. For each pathway, we considered only the paired samples with pathway coverage greater than 0.8 in either or both Pre and End time points and applied Wilcoxon signed-rank tests. We calculated the fold change as the log_2_ of the ratio between the mean pathway abundance at End and at Pre. Nominal *P* values were corrected for multiple hypothesis testing using the Benjamini–Hochberg method (43). FDR-corrected *P* values were reported as *q* values. Overall, we tested 63 pathways for RS2, 61 for Ctl, and 62 for RS4. We focused on pathways with *q* values <0.2 and abs(LFC) >0.1 (Table S6), and we also computed LFC in each group separately to assess whether the changes were consistently observed in both group A and group B (Table S7).

#### Identification of UniRef50 affected by RS supplementation

We first normalized UniRef50 gene family abundances provided by HUMAnN with CoPM. As for pathways, we analyzed samples from group A and B together, and for each intervention, we considered subjects with both Pre and End samples available (RS2, *n* = 47; Ctl, *n* = 44; and RS4, *n* = 44). We tested the UniRef50 detected in at least 10 individuals and with mean CoPM ≥2 both before and after each intervention (RS2, *n* = 86,219; Ctl, *n* = 93,480; and RS4, *n* = 88,519). As described above, we applied Wilcoxon signed-rank tests between paired samples followed by multiple hypothesis correction of *P* values (Benjamini–Hochberg method). We then calculated the fold change (log_2_ of the ratio between mean CoPM at End and mean CoPM at Pre). We focused on UniRef50 gene families with *q* values <0.01 and abs(LFC) >1, identifying those significantly increased (RS2, *n* = 1,134; Ctl, *n* = 0; and RS4, *n* = 1,741) or decreased in each intervention (RS2, *n* = 112; Ctl, *n* = 0; and RS4, *n* = 43). To assign label descriptions to UR50, we mapped them against the UniRef50 db (v201901). For RS2, 741/1,134 (65.3%) increased and 39/112 (34.8%) decreased UR50 were successfully annotated; for RS4, 863/1,741 (49.6%) increased and 17/43 (39.5%) decreased. In the volcano plots in Fig. S5a and b, we highlighted the UR50 with names containing “amylase,” “glycosidase,” “glycoside hydrolase,” “pullulanase,” “starch,” and “Sus.” To identify UniRef50 gene families commonly increased with both RS2 and RS4, we relaxed significance fold change thresholds to *q* values <0.05 and LFCs >0.5, resulting in 34 UR50, 27 of which could be mapped and included a glycogen debranching enzyme, a glycoside hydrolase family 2, an α/β hydrolase, and an α-mannosidase.

#### Identification of CAZymes affected by RS supplementation

We considered the column uniq_rpkm from the Cayman output (25). We analyzed samples from group A and B together, and for each intervention, we considered subjects with both Pre and End samples available (RS2, *n* = 47; Ctl, *n* = 44; and RS4, *n* = 44). We tested the CAZymes with at least 5% prevalence and mean rpkm ≥2 either at Pre or End or at both time points (RS2, *n* = 312; Ctl, *n* = 305; and RS4, *n* = 313). We applied Wilcoxon signed-rank tests between paired samples, and we corrected *P* values for multiple hypothesis testing (Benjamini–Hochberg method), and we then calculated the LFC (log_2_ of the ratio between mean CoPM at End and mean CoPM at Pre). Finally, we focused on CAZymes with a *q* value of <0.05 and abs(log_2_FC) of >0.5 in each intervention (Table S9).

For the HUMAnN-based analysis, we first normalized gene families’ abundances provided with CoPM. Then we regrouped the UniRef90 gene family table using the script humann_regroup_table and a custom mapping file to get CAZymes. Then, we performed differential abundance analysis as described for the Cayman-based analysis. The tested CAZymes were RS2 (*n* = 86), Ctl (*n* = 85), and RS4 (*n* = 89).

## Data Availability

Metagenomic reads are publicly available in NCBI-SRA under accession number PRJNA1402699.

## SUPPLEMENTAL MATERIAL

Supplemental figures spectrum.00763-26-s0001.docx Figures S1 to S5.

Supplemental tables spectrum.00763-26-s0002.xlsx Tables S1 to S9.

ASM does not own the copyrights to Supplemental Material that may be linked to, or accessed through, an article. The authors have granted ASM a non-exclusive, world-wide license to publish the Supplemental Material files. Please contact the corresponding author directly for reuse.
