# Supplementary material for: Resistant starch types 2 and 4 induce distinct and reversible changes in the human gut microbiome
Source: Microbiol Spectr. 2026 Jul 24;14(9):e00763-26. doi: 10.1128/spectrum.00763-26 (PMC13532264; doi:10.1128/spectrum.00763-26)
Supplement: Supplementary file 1 [file spectrum.00763-26-s0001.docx]

**Supplementary Figure 1. Participants characteristics at baseline.
a) No significant differences in Body Fat percentage and Age between the two study groups at baseline.** Study participants were randomly allocated to either Group A or B (**Methods**), resulting in no Baseline differences in body fat and age between the two groups. **b) Participants tend to maintain their habitual diet throughout the study.** For each sample, dietary macronutrient intakes (i.e., carbohydrates, fibers, proteins, fats, sugar) were estimated based on food records (**Methods**) and adjusted by energy (g/1,000 kcal/day), and then scaled to sum to 1. Pairwise Euclidean distances were computed between intake records. The green box plots depict distances between records of the same subject, while the orange box plots are from different subjects. Dietary records of the same subject are significantly more similar than those collected from different subjects. Interestingly, as the study advances (i.e., from Baseline (Bsl) to the last intervention (Trt3)), dietary intake progressively diverges. The boxplots show the first and third quartiles (boxes) and the median (middle line); whiskers extend to 1.5× the interquartile range (IQR).

**Supplementary Figure 2. Alpha and Beta diversity.
a) tSNE of beta diversity between samples at the various timepoints.** We calculated the Bray-Curtis beta diversity between sample pairs and performed a tSNE dimensionality reduction for plotting in two dimensions. Each point represents a sample. We also performed PERMANOVA analyses (**Methods**) between group A and group B at each timepoint. While we found no significant difference between the two groups at baseline, at all pre-intervention timepoints (Pre Int. #1, #2, #3), and post-control intervention timepoint (End Int. #2) (all *p* values > 0.5), we observed a significant shift at End Int. #1 (*p* value = 2.97e-2) and at End Int. #3 (*p* value = 9.90e-3). **b) Alpha diversity.** Shannon’s diversity index showed a significant reduction after supplementation with RS type 2, especially in Group B, while species Richness was not affected by the interventions. The boxplots show the first and third quartiles (boxes) and the median (middle line); whiskers extend to 1.5× the interquartile range (IQR). **c) tSNE of beta diversity between samples for each intervention.** For each treatment (RS2 in blue, Control (Ctl) in green, and RS4 in yellow), we combined samples from the two groups and included only subjects with samples both at “Pre” and “End” timepoints, respectively depicted with light and dark colors. The PERMANOVA analyses revealed a significant difference in beta diversity between the Pre and End of each intervention with RS (RS2: *p* value = 4.95e-2; RS4: *p* value=3.96e-2), but not with the Control intervention (*p* value=1).

**Supplementary Figure 3. Impact of RS2 and 4 supplementation on group A and B.
a) Consistent results were observed by analyzing the two groups separately or together.** The volcano plots highlight the SGB significantly increasing and decreasing upon RS2 (upper row) and RS4 (lower row) supplementation, with samples from Group A and B analyzed separately (respectively Blue and Orange header) or together (Combined: Gray header). SGBs increasing or decreasing in the Combined analyses (**Fig. 2a**) are marked respectively with blue and red, with light colors used for the SGBs only significant in the Group-specific analyses. The x axis represents the log_2_ fold change between the relative abundance before and after each intervention. The vertical grey dotted lines mark SGBs with an abs(log_2_FC) >1. The y axis reports the log_10_ of the *q* values (*p* values were corrected for multiple hypothesis testing using the Benjamini-Hochberg method). The horizontal grey dotted line marks SGBs with a *q* value < 0.2 for the first two columns (Groups separated) or *q* value < 0.05. **b) The impact of RS supplementation on “negatively-affected” SGBs is not RS-type-specific.** The line plots depict the overall relative abundance throughout the study timepoints of the four SGBs which experienced the strongest decrease in relative abundance (Log_2_FC < -1.5 and lowest *q* values) upon the two RS interventions (first two for RS2 and last two for RS4). Individuals assigned to Group A (RS2 at intervention #1 and RS4 at intervention #3) are depicted in blue, while those of Group B in orange (RS4 at intervention #1 and RS2 at intervention #3). In the figure, all Pre and End metagenomics samples are included with corresponding baseline samples. (**Methods**).

**Supplementary Figure 4. Strain retention and subject-specific variability in response to RS intake.
a) The same strains persist throughout the 7-weeks intervention period.** Same-individual (cyan) and different individual (purple) genetic distance comparisons for the SGB significantly increasing upon RS supplementation (**Fig. 2**). Only participants in which the SGB strain-level profiling was available for more than one sample are kept for the comparisons. Only SGBs with comparisons for more than 10 different participants are reported, the *n* in each histogram corresponds to the number of distinct individuals. The x axis reports the centred normalised phylogenetic distance (nGD), in which phylogenetic distances are divided by the median distance among samples from different individuals. Strain identity thresholds were set 0.1 and are marked by the grey dashed line. **b) The strain-level phylogenetic tree of *Ruminococcus bromii*.** Each individual retains the same strain across longitudinal samples. Node colors represent samples from distinct subjects, only subjects with at least two samples included in the StrainPhlAn tree are shown. The inner ring indicates the study phase, and the outer ring displays the arcsin transformed relative abundance. **c) Individual SGBs log_2_FC in response to RS4 and RS2 intake.** Abundance fold change patterns for the SGBs with a significant increase at the cohort level following RS4 (upper) and RS2 (lower) supplementation (**Fig. 2**; *q* value < 0.05 and log_2_ (Mean ab. End) / (Mean ab. Pre) >1). With only a few exceptions (e.g., subject ID 326), we observed that these SGBs were consistently upregulated among subjects. **d)** **The strain-level phylogenetic tree of *Bifidobacterium adolescentis*.** The phylogeny of *B. adolescentis* shows strain-specific patterns in response to RS2 (left) and RS4 (right) supplementation. While some subjects experience an increase in *B. adolescentis* abundance (log_2_FC > 0 marked in green) from Pre (lighter blue) to End (darker blue) RS2 (or RS4) intake, others experience a decrease (log_2_FC < 0 marked in red), indicating possible strain-specific abilities to utilize RS. Each node color represents samples from a different subject. Only subjects with either “Pre RS2” or “End RS2” and “Pre RS4” or “End RS4” samples included in the tree were plotted (**Methods**). Seven reference genomes from strains previously tested for resistant starch utilization in vitro were included in the tree (**Methods**). Strains P2P3, L2-32, 22L, DSM24849 have been reported to utilize RS (marked with the green stars); strains 703B, DSM20086 to not use RS (red stars); while DSM20087 exhibited low RS utilization (orange star).

**Supplementary Figure 5. UniRef50 gene families and CAZymes (HUMAnN-based analysis) significantly altered by RS intake.
a-b)** Volcano plots of the UniRef50 gene families affected by RS2 (*q* value < 0.05, *n*=14,935) **(a)** and RS4 supplementation (*q* value < 0.05, *n*=10,557) **(b)**. We colored UniRef50 with *q* value < 0.01 and emphasized (with darker and bigger circles) those annotated as enzymes known to be involved in resistant starch metabolism, such as amylases, glycosidases, glycoside hydrolases, pullulanases, starch-utilization system, and starch-binding proteins.

**c)** Fold change and significance of the CAZymes across interventions (RS2: blue squares, Ctl: green dots, RS4: yellow diamond) from the HUMAnN output. While no significant differences were observed in RS2 and Ctl, RS4 intake resulted in thirteen significantly increased CAZymes, including nine glycoside hydrolase families.
